# A case of endonasal balloon‐assisted dacryoplasty after failure of endonasal dacryocystorhinostomy

**DOI:** 10.1002/ccr3.2956

**Published:** 2020-05-19

**Authors:** Pietro Indelicato, Alessandro Vinciguerra, Antonio Giordano Resti, Matteo Trimarchi

**Affiliations:** ^1^ Division of Head and Neck Department Otorhinolaryngology unit IRCCS San Raffaele Scientific Institute Milano Italy; ^2^ School of Medicine Vita‐Salute San Raffaele University Milano Italy; ^3^ Division of Head and Neck Department Ophthalmologic unit IRCCS San Raffaele Scientific Institute Milano Italy

**Keywords:** ballooning, DCP, nasolacrimal duct obstruction, revision surgery

## Abstract

Endonasal balloon‐assisted dacryoplasty is a minimally invasive technique that uses a high‐pressure balloon catheter introduced into the lacrimal pathway through the nasal fossa into the neorhinostomy to treat recurrent epiphora after primary dacryocystorhinostomy. This procedure can be considered to be a reliable technique in patients unfit for general anesthesia.

## Graphical abstract

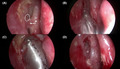

## 1 INTRODUCTION

Epiphora is a common clinical condition that affects more than 3% of all adult ophthalmologic patients, which is generally caused by obstruction of the nasolacrimal duct (NLDO, nasolacrimal duct obstruction).^1^ Dacryocystorhinostomy (DCR), a surgical procedure used to create a fistula that bypasses the site of obstruction and restores the tear flow, is widely used to treat NLDO.

The procedure can be executed by adopting either an external or an endoscopic endonasal approach. At present, the gold standard method for primary NLDO is external DCR (EXT‐DCR) with a success rate between 90%‐95%.^2^, ^3^ Recently, endoscopic DCR (END‐DCR) has been suggested by many authors to be an alternative to the external approach, offering high rates of success (75%‐97%)^4^, ^5^ and reduced postoperative complications.^6^, ^7^ Both END‐DCR and EXT‐DCR fail in up to 10% of patients, leading to inefficient drainage of tears and relapse of epiphora.^8^, ^9^, ^10^ The presence of membranous obstruction of the nasal ostium, formation of granuloma or synechiae next to the neorhinostomy, and lacrimal pump disruption are the most common causes of surgical failure.^11^, ^12^

Many treatments are available in case of recurrent epiphora after primary surgery, including EXT‐DCR, END‐DCR, balloon dacryoplasty, and observation, which can be considered the best choice in slightly symptomatic elderly patients.^13^ Secondary EXT‐DCR and END‐DCR are the most widely adopted options; however, these techniques are associated with a prolonged healing process, which may induce further fibrosis and worsen stenosis.^14^

Balloon‐assisted dacryoplasty (DCP) is a less invasive procedure that is performed using a high‐pressure balloon catheter introduced into the lacrimal system passing through the superior or inferior canaliculus. The aim of this procedure was to enlarge the site of the stenotic lacrimal tract, which is typically located in proximity to the neorhinostomy.^15^ Since a transcanalicular approach can cause damage to the canalicular system,^16^ endoscopic endonasal DCP has been considered as an alternative approach in revision cases with the aim of restoring physiological lacrimal drainage. In fact, such DCP technical variation allows the enlargement of the neorhinostomy passing through the nasal neorhinostomy avoiding any potential lesion of the canalicular system.

Herein, we present the case of a female patient who underwent transnasal balloon‐assisted DCP after failure of primary endoscopic DCR.

## 2 CASE REPORT

A 78‐year‐old woman complained of relapsing right‐side epiphora and recurrent dacryocystitis after failure of an END‐DCR performed 3 months before. Symptoms appeared immediately after surgery, even though the procedure was carried out without any intraoperative complications. On the 15 day of postoperative follow‐up, endoscopic endonasal examination demonstrated the presence of a sinonasal synechiae next to the neorhinostomy, determining a near‐total obstruction of the ostium created by the previous surgical procedure.

### 2.1 Investigation and treatment

Lacrimal probing and irrigation were performed, by an ophthalmologist, and led to upper lacrimal punctum liquid spillage and total absence of lacrimal flow through the neorhinostomy. Surgical reintervention was deemed necessary. Considering the patient's age, comorbidities (type II diabetes, hypertension, chronic bronchitis, and chronic kidney disease), presence of partially healed mucosa, and the recent surgery, we decided to adopt a less invasive endoscopic approach using a balloon catheter introduced through the nose to enlarge the stenotic neorhinostomy.

**FIGURE 1:**
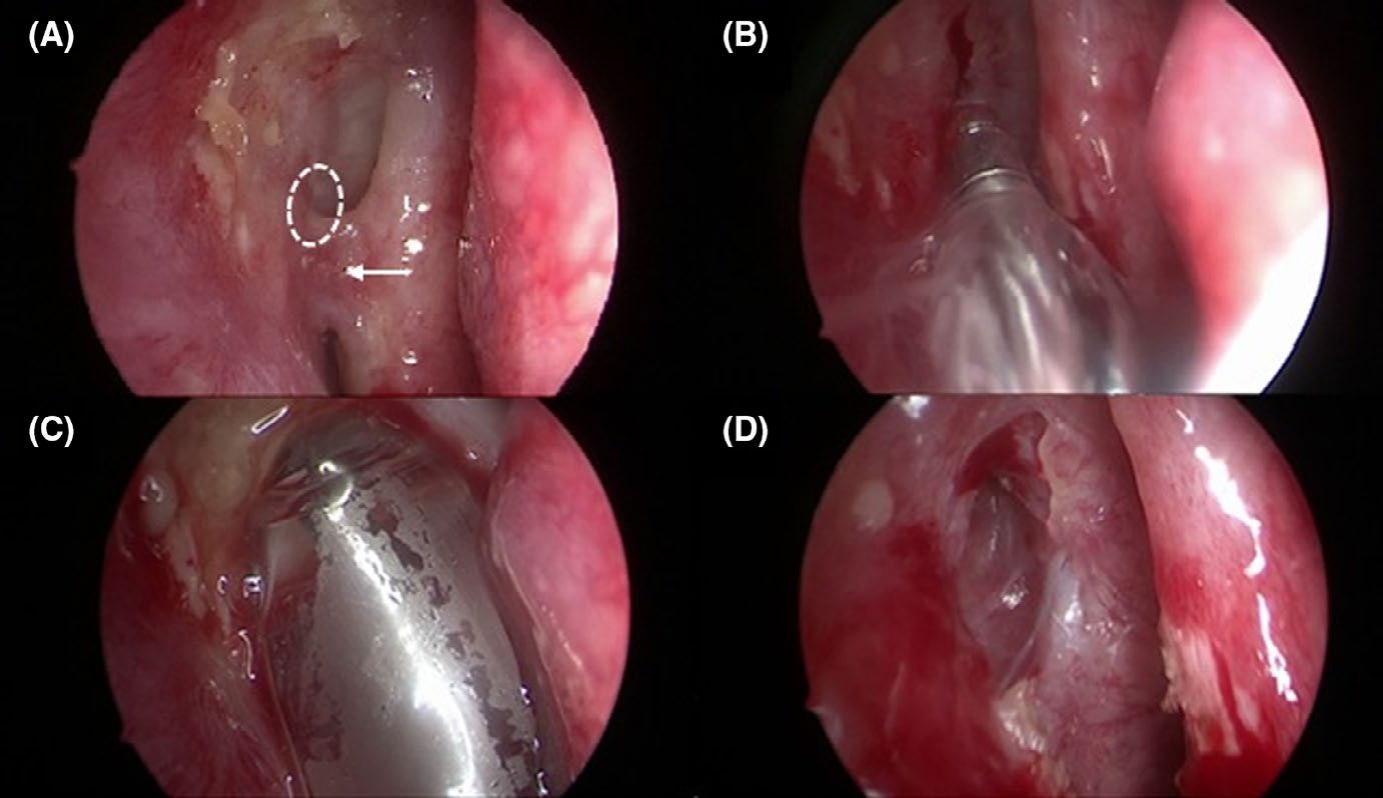
Endoscopic endonasal balloon DCP: A, Sinonasal sinechiae (white arrow) giving rise to subtotal stenosis of neorhinostomy (dashed white circle); B, Transnasal introduction of the deflated balloon catheter next to the previous stenotic neorhinostomy; C, Complete balloon catheter inflation; D, Enlarged neorhinostomy

**FIGURE 2:**
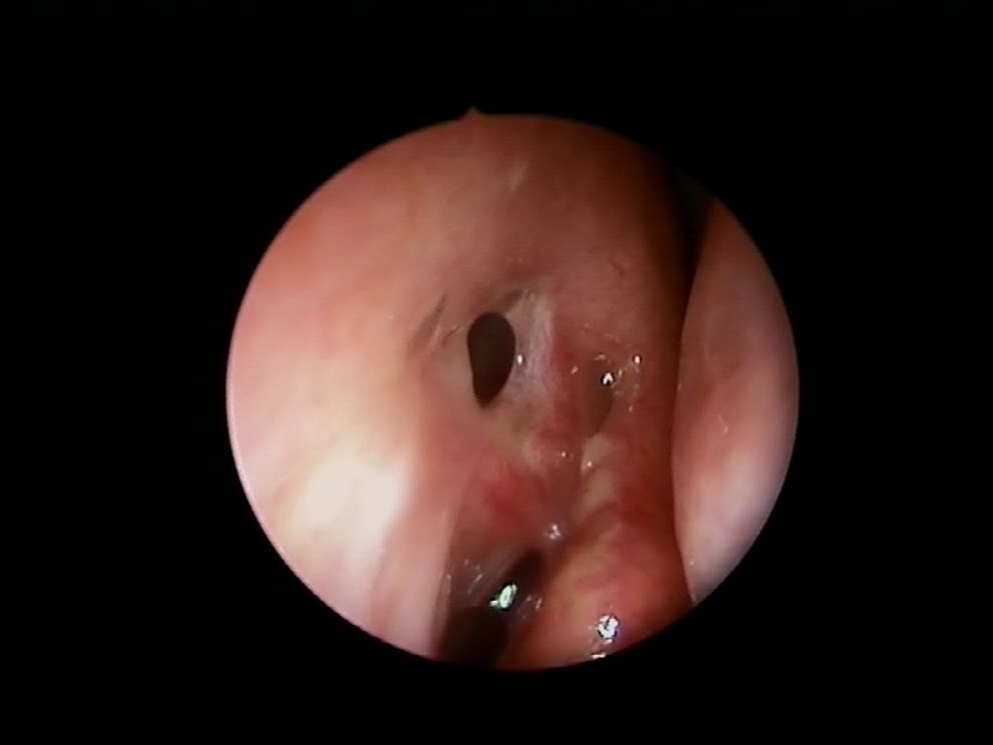
Postoperative follow‐up at day 45 after surgery

Surgery was performed under general anesthesia. After endoscopic lysis of the sinonasal synechiae, the lacrimal punctum was dilated by an ophthalmologist with a lacrimal probe inserted into the canalicular system down the stenotic lacrimal tract. The tip of this probe was used as a guide to permit direct endoscopic endonasal visualization of the stenotic ostium. An 8 mm balloon catheter (XprESS™ LoProfile ENT dilation system, Entellus medical^®^) was introduced under endoscopic guidance into the nasal neorhinostomy created during primary DCR passing through the nasal cavity. After connection of the catheter to the inflation device, the rhinostomy was enlarged by the balloon system, which was inflated to 12 atm for 20 seconds and then deflated for 10 seconds. The balloon was then reinflated to 8 atm for 20 seconds and deflated for 10 seconds. This last cycle was repeated twice. After completing 3 cycles, the balloon catheter was completely deflated and carefully removed from the nasal neorhinostomy (Figure 1). The entire surgical procedure lasted 18 minutes. Postsurgical nasal packing was not necessary given the absence of active bleeding after the procedure. The patient received postoperative oral antibiotics (amoxicillin + clavulanate), was prescribed nasal washes, and used emollient local ointment and antibiotic‐steroid eye drops for a 7‐day period. At 45 days after the procedure, the neorhinostomy was still open (Figure 2); there was no evidence of recurrent epiphora at 5 months after surgery.

## 3 DISCUSSION

Nasolacrimal duct obstruction is a common condition observed in ophthalmology practice that can be managed both surgically and nonsurgically. The obstruction can be distinguished as congenital or acquired. Primary acquired nasolacrimal duct obstruction is determined in most cases by idiopathic fibrosis or inflammation, while infection, traumatic events, surgery, or neoplasms may cause secondary acquired NLDO.^17^, ^18^, ^19^, ^20^, ^21^, ^22^

Many surgical treatments are available for NLDO, but there is a lack of consensus in the literature about the best procedure in primary and revision cases. Dacryoplasty is a minimally invasive technique in which a balloon catheter is used to dilate a stenotic lacrimal tract.^23^ Although a transcanalicular approach has been described, there are no reports on the use of transnasal balloon‐assisted DCP after failure of endoscopic primary DCR. This minimally invasive treatment can provide high quality results with shorter surgical time: in fact, both anatomical and functional success was achieved. Anatomical success was defined when a patent ostium on irrigation was achieved; functional success was defined as free lacrimal flow on functional test and subjective resolution in tearing symptoms.

Both transnasal and transcanalicular DCP present several advantages over EXT‐DCR or END‐DCR. First, differently from EXT‐DCR these procedures do not require a skin incision and both preserve the orbicularis muscle, which contributes to lacrimal pump function.^24^ Second, minimally invasive surgery reduces postoperative complications such as nasal bleeding and formation of nasal synechieae.^25^ Lastly, the reduced operating time makes DCP a feasible procedure in patients who cannot tolerate prolonged general anesthesia. Considering transcanalicular and endonasal DCP, the latter may prevent potential canalicular damage by avoiding manipulation of the proximal lacrimal drainage system, leading to a safer procedure.^16^

Furthermore, an endoscopic endonasal approach consents creation of a wider neorhinostomy by using 5, 8, or 9 mm diameter balloon catheters.

To our knowledge, this is the first case report describing the use of transnasal ballon‐assisted DCP after a failed primary DCR; for this reason, the comparison of our procedure with other studies is difficult. Lee et al reported on the use of balloon DCP after failed EXT‐DCR in three patients with sarcoidosis.^26^ The two early failures (at 3 months) were successfully treated with DCP, and both patients were asymptomatic at 42 and 5 months, respectively; in the case of late failure (47 months), the same procedure was performed without improvement of symptoms. However, the authors did not specify either the surgical approach adopted (transcanalicular vs transnasal) or the parameters considered to define successful treatment (anatomical vs functional), making these results incomparable. Conversely, Lee et al described 18 cases who underwent transcanalicular balloon‐assisted revision DCP after END‐DCR with anatomical and functional success rates of 84% and 74%^13^ and a follow‐up of 11 and 19 months, respectively.^13^ A silicone stent was inserted into the lacrimal drainage system and left in place for 4‐6 weeks in all patients.

There are controversial reports in the literature regarding the benefits of silicone stenting. Several authors sustain that silicone tubing may improve surgical outcomes by maintaining the patency of the lacrimal system.^27^, ^28^ At the same time, multiple systematic reviews and meta‐analyses have not demonstrated any additional advantages in silicone device placement, especially in endoscopic procedures.^29^, ^30^ A silicone stent may act as foreign inorganic material, leading to granulation with subsequent closure of the rhinostomy.^31^ Considering the presence of a pre‐existing neorhinostomy and the low invasiveness of our procedure, we retained it unnecessary to place a lacrimal stent.

In addition to silicon stenting, other adjunctive treatments have been reported to improve surgical outcomes and prevent early failure. Application of corticosteroids and mitomycin C (MMC) at the site of neorhinostomy can potentially reduce scarring through action on the inflammatory and proliferative phases of wound healing, respectively. Zeldovich et al reported a success rate of 89% in a prospective, nonrandomized series of 16 patients undergoing revision END‐DCR, in which betamethasone was injected into the lacrimal sac and scar tissue surrounding the ostium.^32^ Similar results (93% success rate) were obtained by Li et al in a retrospective case series of 69 END‐DCR followed by local application of triamcinolone soaked gelfoam.^33^ Despite promising results, no large randomized control trials have been performed on the use of corticosteroids as adjuvant therapy in revision surgery.

Conversely, many studies have been carried out on the efficacy of intraoperative MMC in EXT‐DCR and END‐DCR, although the results have not always been consistent.^34^, ^35^, ^36^, ^37^ Mitomycin C is an antineoplastic drug derived from *Streptomyces caespitosus* that inhibits the synthesis of DNA, RNA, and protein by through inhibition of collagen synthesis by fibroblasts.^38^ A systematic review and metanalysis by Cheng et al^39^ reported that application of MMC in a subgroup of patients undergoing EXT‐DCR was more likely to keep patency of irrigation during follow‐up, while in a subgroup of patients who underwent END‐DCR, the difference was not significant. Since there is no unanimous consensus regarding the use of corticosteroids and MMC after DCR – DCP, we do not use these adjunctive agents in our surgeries.

Finally, the use of postoperative antibiotic therapy is still questioned and there is no evidence that regular administration of oral antibiotic after surgery may improve surgical outcomes. Lehmann et al in a retrospective study on 596 cases showed that patients who failed primary END‐DCR were more likely to have postoperative complications, including infections; in addition, the occurrence of a postoperative complication was significantly associated with primary END‐DCR failure.^40^, ^41^ Since operative site infection may result in scar formation and compromise healing of a patent lacrimal system, we administer postoperative antibiotic therapy.

In conclusion, transnasal balloon‐assisted revision DCP can be considered as a valid, safe, and minimally invasive surgical procedure to treat recurrent epiphora after failure of endoscopic or external DCR. The procedure should be considered as a feasible option in the case of recurrent epiphora after multiple surgical procedures or in patients who cannot undergo more aggressive treatment or prolonged general anesthesia due to age and comorbidity. The main advantages of this surgery are shorter surgical time and reduced postoperative complications.^13^, ^26^

## CONFLICT OF INTEREST

The author(s) declare no potential conflicts of interest with respect to the research, authorship, and/or publication of this paper.

## AUTHOR CONTRIBUTIONS

PI: drafted the article and made substantial contributions to acquisition of data; AV: made substantial contributions to acquisition of data and revised the manuscript critically for important intellectual content; AGR: made substantial contributions to acquisition of data; MT: conceptualized and designed the study, made substantial contributions to conception and acquisition of data, critically reviewed the manuscript for important intellectual content, and approved the final version.
